# Scaling active spaces in simulations of surface reactions through sample-based quantum diagonalization

**DOI:** 10.1038/s41598-026-58228-0

**Published:** 2026-06-22

**Authors:** Marco Antonio Barroca, Tanvi P. Gujarati, Vidushi Sharma, Rodrigo Neumann Barros Ferreira, Young-Hye Na, Maxwell Giammona, Antonio Mezzacapo, Benjamin Wunsch, Mathias B. Steiner

**Affiliations:** 1 IBM Research, Rio de Janeiro, 20031-170 RJ Brazil; 2 IBM Quantum, IBM Research - Almaden, San Jose, 95120 CA USA; 3 IBM Research, IBM Research - Almaden, San Jose, 95120 CA USA; 4 IBM Quantum, IBM T.J. Watson Research Center, Yorktown Heights, New York, 10598 NY USA; 5 IBM Research, IBM T.J. Watson Research Center, Yorktown Heights, New York, 10598 NY USA; 6 Centro Brasileiro de Pesquisas Físicas, Rio de Janeiro, 22290-180 RJ Brazil

**Keywords:** Quantum computing, Quantum chemistry, Quantum embedding, Materials science, Chemistry, Materials science, Mathematics and computing, Physics

## Abstract

Quantum-chemical simulations are essential for predicting energies of chemical reactions. Accurately solving the many-body Schrödinger equation for reagent and product states of most relevant chemical processes is, however, unfeasible. Quantum computing offers a pathway for predicting energies of correlated electronic systems with localized interactions. Here, we apply a quantum embedding approach for investigating oxygen reduction reactions at the electrode surface in Lithium batteries, a representative example of energetic analysis in localized chemical reactions. We employ an active space selection method based on density difference analysis for identifying the orbitals involved in the reaction. Leveraging the Local Unitary Cluster Jastrow ansatz for state preparation, the active-space orbitals are then processed on a quantum computer. As quantum algorithms, we use Sample-based Quantum Diagonalization, SQD, and its extended version, Ext-SQD, which integrates electronic excitations into the quantum-selected electronic configuration subspace. The largest configurations are represented by quantum circuits mapped onto 80 qubits of an IBM Heron R2 quantum processing unit. For up to 12 orbitals, we are able to benchmark the quantum-computed reaction energies against results obtained with Complete Active Space Configuration Interaction. For benchmarking results in active spaces as large as 32 orbitals, we resort to Heat-Bath Configuration Interaction and Coupled Cluster Singles and Doubles calculations, respectively. At 27 orbitals, the Ext-SQD results exhibit prediction accuracy improvements with regard to the standard, quantum-chemical reference methods that remain computationally feasible at that scale. The results indicate the potential of sample-based quantum diagonalization for performing high-accuracy reaction modeling in chemistry and materials science.

## Introduction

Computational modeling of correlated electronic systems is essential for accurately predicting chemical reaction energies. Exact solutions of the electronic Schrödinger equation are unfeasible, forcing the adoption of approximate methods. Although Density Functional Theory (DFT) is widely used, its exchange-correlation approximations fail to capture strong electron-electron correlations in complex systems^1,2^.

Hybrid quantum-classical approaches, also termed Quantum-centric Supercomputing^3^, offer an alternative pathway by leveraging quantum algorithms that, in principle, simulate electronic wavefunctions more efficiently than classical methods^4–6^. Due to today’s quantum hardware limitations, short-term approaches to practical quantum computing in chemistry integrate quantum algorithms within classical simulation workflows. An important application is the simulation of select active orbital spaces, a technique which reduces the size of the system under study while preserving its characteristic electronic correlations. Demonstrated approaches include Density Difference Analysis (DDA) with coupled-cluster natural orbitals^7^, Atomic Valence Active Space (AVAS) with atomic valence orbitals^8^, and Density Matrix Embedding Theory (DMET) with partitions of correlated fragments^9^.

The Variational Quantum Eigensolver (VQE)^10–12^ method has been explored for predicting molecular ground states as well as reaction energies. However, challenges in ansatz design, the occurrence of barren plateaus and a high sampling penalty have posed practical limitations to the size of the chemical system under study^13–16^. Recently, Sample-Based Quantum Diagonalization (SQD) was successfully applied to project an electronic Hamiltonian onto a reduced subspace constructed from a sampled set of electronic configurations, before processing the quantum-selected subspace with classical diagonalization techniques^17^. An enhanced version of this method, Extended SQD (Ext-SQD), introduces excitation operators to refine the configuration space and improve the accuracy of energy predictions^18^. In the SQD framework, a process termed configuration recovery plays a crucial role. By iteratively correcting the sampled electronic configurations with regard to electron number and symmetry constraints, the recovery step validates the configurational outcomes.

For constructing a reduced subspace to be used with SQD and Ext-SQD, the electronic configurations must be efficiently sampled with a quantum processing unit. The Local Unitary Cluster Jastrow (LUCJ) ansatz provides a shallow, hardware-compatible circuit construction while retaining important electronic correlations. Initializing circuits with coupled-cluster parameters taken from “classical”, i.e., quantum-chemical simulation methods performed with standard computers, further enhances the quality of selected configurations^19^. The approach enables accurate predictions of ground-state and excited-state energies, allowing for the quantum-assisted predictions to be scaled up and to be benchmarked against established reference methods of quantum chemistry^17–20^.

In this work, we apply an advanced, hybrid quantum-classical method based on SQD and Ext-SQD for predicting the ground state energy difference of reagent and product states in molecular surface reactions. We increase stepwise the orbital count in the active space and sample the most relevant electronic configurations by means of a customized LUCJ circuit on a pre-fault tolerant quantum device. This way, we are able to benchmark the prediction accuracy obtained with the quantum-sampled configurations against the results obtained with high-accuracy, quantum-chemical simulations performed on standard computers.

Specifically, we show that incorporating excitation operators into the SQD-selected subspace improves prediction accuracy by enriching the effective configuration space, while introducing a classical post-processing overhead that remains manageable for the systems and excitation-selection parameters considered here. Using a scalable SQD workflow, we execute large circuits of up to 80 qubits on ibm_aachen, a quantum computer equipped with a Heron R2 processing unit containing 156 qubits. Starting from DFT-based atomic geometries and energies, we find that Ext-SQD matches and, at larger active spaces, eventually outperforms classical simulation methods. To our knowledge, this is the first application of SQD and Ext-SQD in chemical reaction predictions. Finally, we discuss the implications of our study and outline next steps to further refine and scale quantum-assisted simulations in chemistry and materials science.

## Results

### Surface reaction

The reaction of lithium and oxygen in Li-metal anode batteries plays a crucial role in stabilizing the solid electrolyte interphase (SEI) and enhancing battery performance. While offering high energy density, Li-batteries face significant safety challenges due to dendrite formation, which can lead to short circuits and failure. The inclusion of dissolved oxygen in the electrolyte has been shown to mitigate dendrite growth by promoting the formation of lithium oxides, thereby improving stability and cycle life^21^.

The primary reaction of interest in these systems is

$$\begin{aligned} {4 \hbox {Li} + \hbox {O}_{2} -> 2 \hbox {Li}_{2}\hbox {O}}, \end{aligned}$$

resulting in the formation of lithium oxide ($\hbox {Li}_{2}$O), which is deposited at the SEI. However, depending on local conditions such as oxygen concentration and electrolyte composition, other lithium oxides, including lithium peroxide ($\hbox {Li}_{2}\hbox {O}_{2}$) and lithium superoxide ($\hbox {LiO}_{2}$), may also form. The competition between these different reaction pathways is a critical factor influencing battery performance and longevity^22,23^.

Previous studies have proposed reaction mechanisms for various surface-mediated electrochemical processes^24–26^. Following a similar approach, we investigate lithium–oxygen surface energetics through quantum chemistry calculations. In lithium–oxygen systems, lithium oxidation and oxygen reduction can lead to the formation of $\hbox {Li}^{+}$ and surface-bound oxygen species, which are relevant to the early stages of lithium oxide formation. A simplified representation of this chemistry is

$${\hbox {Li} + \hbox {O}_{2} -> \hbox {Li}^{+} + {\hbox {O}_{2}}^{-}\hbox {(ads)}}.$$

In this work, however, we do not attempt to resolve the full reaction mechanism explicitly.Instead, we consider the formation of lithium oxide through a comparison between the specific reactant and product surface structures shown in Figure 2a. The reactant geometry corresponds to the optimized initial lithium-surface/O$\vphantom{0}_2$ adsorbate configuration, while the product geometry corresponds to the optimized final surface-bound lithium–oxygen configuration. The reported reaction energies are computed from the ground-state energies of these optimized structures.

The adsorption of $\hbox {O}_{2}$- plays a crucial role in the initial stages of lithium oxide formation. The stabilization of the anions on the surface dictates the pathway for further oxide growth, ultimately influencing the electrochemical performance of the battery^22,27,28^.

**Fig. 1:**
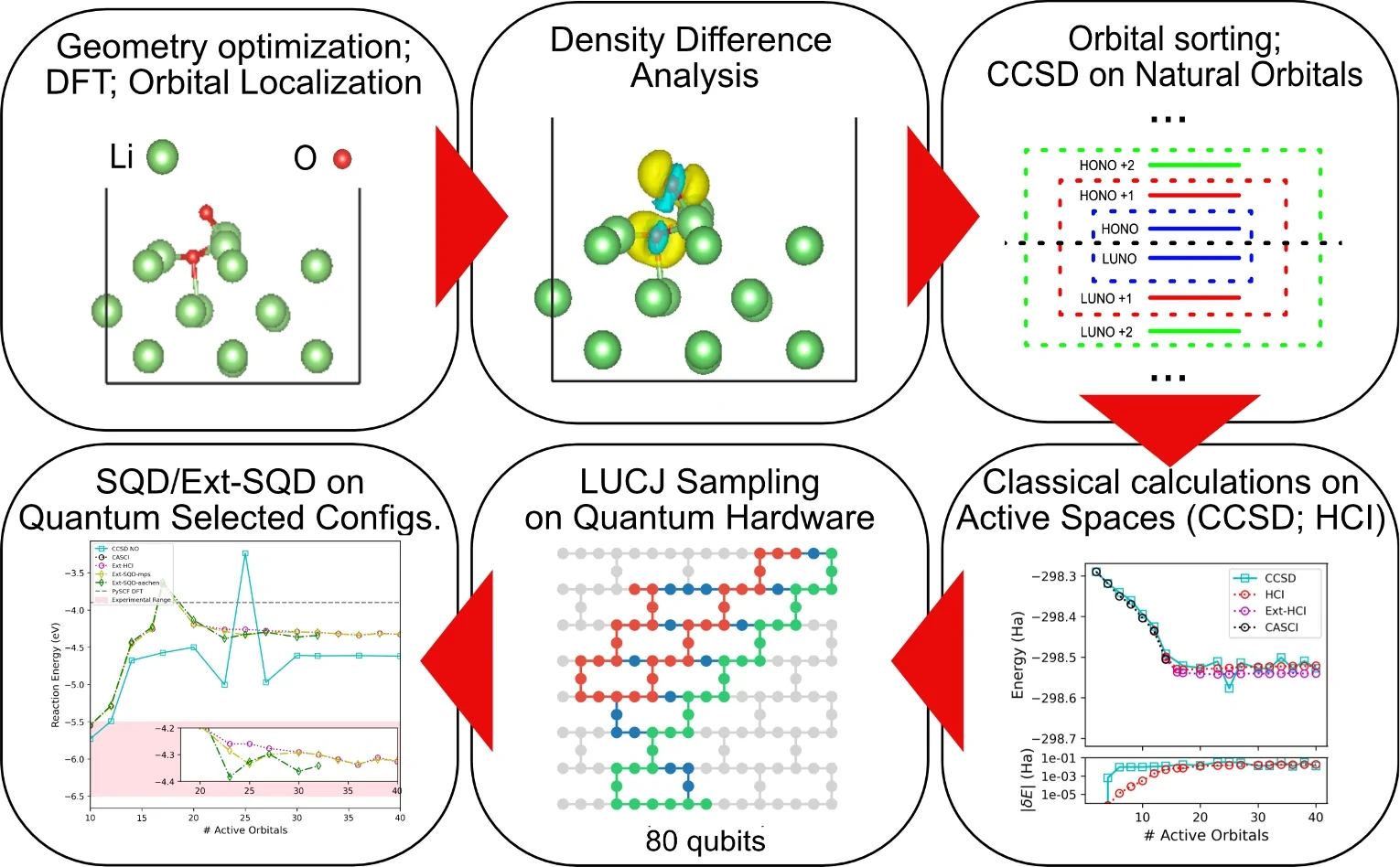
Hybrid, quantum-classical framework for studying lithium oxide formation in surface reactions. Geometry optimization and DFT calculations are followed by active space selection via DDA for natural orbitals, and a LUCJ ansatz samples configurations from quantum hardware. Finally, SQD/Ext-SQD algorithms are used to compute the reaction energies $\Delta E = E_{\text {prod}} - E_{\text {reac}}$.

To study the process, we have designed a computational workflow consisting of geometry optimization, active space selection, and quantum experiments. The workflow, summarized in Figure 1, enables a systematic investigation of lithium-oxygen interactions at the atomic level. The reaction energy is computed as the energy difference $\Delta E = E_{\text {prod}} - E_{\text {reac}}$ between reactant and product states. Predicting the reaction energy allows kinetic analysis of lithium oxide formation, which is essential for battery stability.

### Geometry optimization

All geometry optimizations are performed using DFT as implemented in the Vienna Ab initio Simulation Package (VASP)^29–31^. The structures are treated as periodic in the X-Y plane, with an Li slab thickness of 6.1 Å and a vacuum layer of 12 Å along the Z direction to minimize interactions between periodic images. The Brillouin zone is sampled using a $4 \times 4 \times 1$ **k**-point grid within the Monkhorst-Pack scheme. The oxygen molecule is allowed to relax on the surface of Li slab to ensure an accurate representation of surface interactions. The inert core electrons are accounted for by PAW pseudopotentials, and valence electrons are represented by plane-wave basis set. Only forces and geometries are allowed to relax while cell volume and shape are fixed. The convergence tolerance is set to $10^{-6}~\text {eV}$.

The DFT methodology was chosen to match the two stages of the workflow. Geometry optimization was performed in VASP because a periodic plane-wave description is well suited to the Li slab and adsorbed oxygen geometry, and allows a consistent treatment of the extended surface within periodic boundary conditions. The PAW formalism and the $4 \times 4 \times 1$ Monkhorst–Pack mesh provide a standard description for this slab model. Subsequent energy and density calculations were performed on the optimized geometries in PySCF using the GTH-DZV basis, GTH pseudopotentials, and the PBE functional. Here, PBE was retained for consistency with the geometry-optimization stage, while the PySCF framework is convenient for the localized-orbital, density-difference, and active-space selection analyses used in the remainder of the workflow. For the supercell studied here, the *Γ*-point treatment was found to be sufficient to reproduce the relevant electronic-density features used in this analysis.

In a next step, we utilize the GTH-DZV basis set^32^, in combination with the Perdew-Burke-Ernzerhof (PBE) exchange-correlation functional^33^ and Goedecker-Teter-Hutter (GTH) pseudopotentials^32^, to calculate the energy values and electronic densities for both reactant and product states using DFT within PySCF^34^. The electronic structure calculations are restricted to the *Γ*-point of the supercell as including additional **k**-points does not significantly alter the electronic density.

We find that the predicted reaction energy of lithium oxide at the *Γ*-point, *Δ E = -3.897* eV, is less negative than the literature values, which are typically around *-6* eV^35–38^. This difference is expected, since the literature values we reference are tied to experimental results and simulations that include empirical corrections, whereas the value reported here corresponds to the present *Γ*-point DFT calculation for the specific surface model used in this work.

### Active space selection

The active space selection process combines multiple techniques to systematically identify the most relevant electronic orbitals for quantum simulations. First, Kohn-Sham orbitals are localized using the Pipek-Mezey method to preserve electronic structure characteristics across the Brillouin zone. The localization isolates occupied and virtual orbitals that contribute to the reaction under study. Next, DDA is employed to assess charge redistribution by computing the electronic density difference between the reactant and product states. A tunable threshold function is then applied to filter out orbitals with negligible contributions, ensuring a physically meaningful selection of orbitals for the active space. Seven occupied orbitals are chosen for the reactant and the product as well as all virtual orbitals. The selections are based on the valence space of $\hbox {O}_{2}$ and an initial evaluation of the computed electronic density for the orbitals.

To enhance convergence and accuracy, the active space is further refined using natural orbitals derived from coupled-cluster singles and doubles (CCSD) calculations performed in the initial active space. The selected orbitals are ranked based on occupation numbers, distinguishing those that are fully occupied, unoccupied, or fractionally occupied. In our systems, fractionally occupied orbitals do not occur. The refined active space is then constructed around the Highest Occupied Natural Orbital, HONO, and the Lowest Unoccupied Natural Orbital, LUNO, with additional orbitals included in an “inside-out” fashion for balancing accuracy and computational efficiency as needed.

**Fig. 2:**
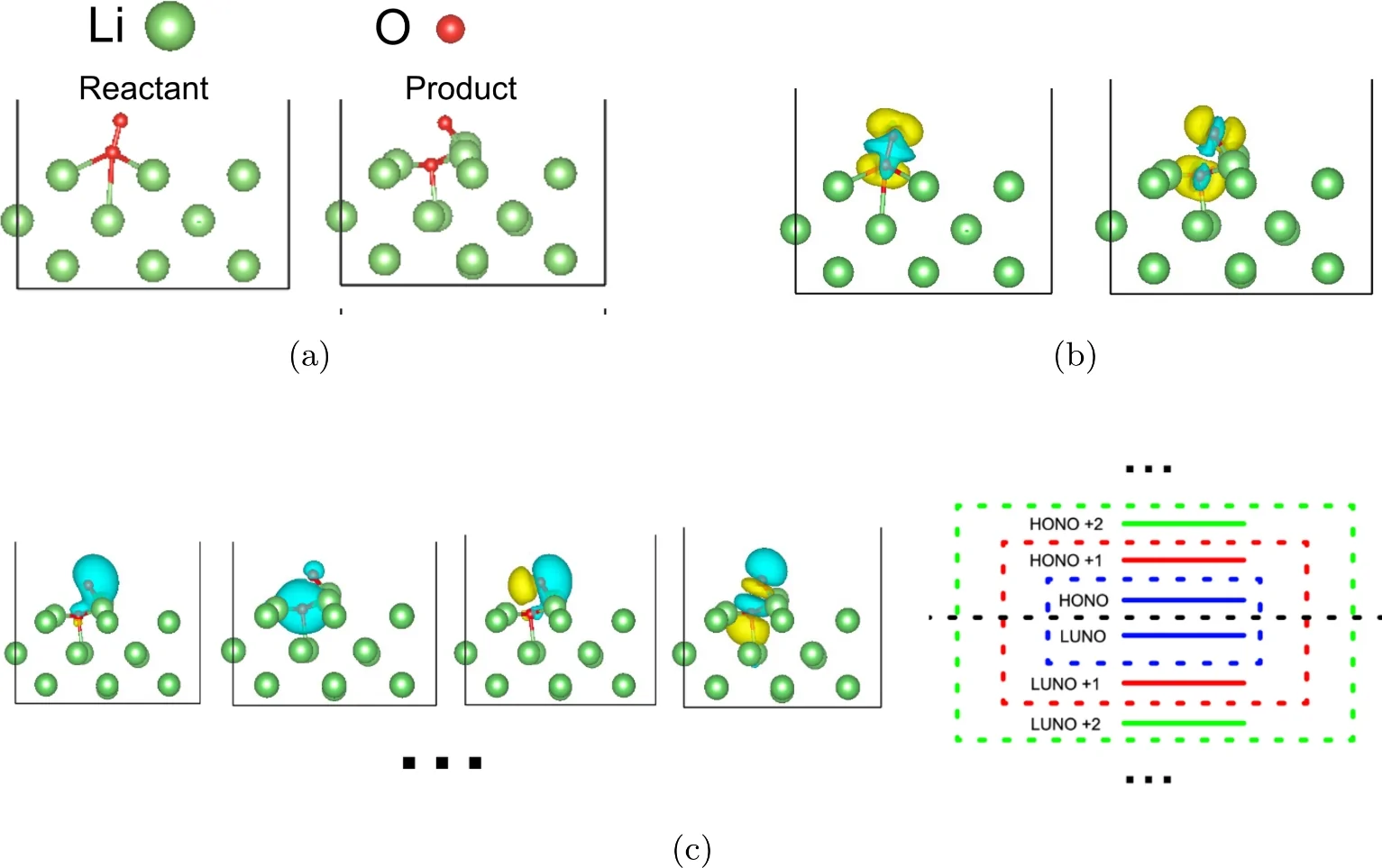
Active-space selection workflow. (**a**) Optimized reactant and product surface geometries defining the initial and final states of the reaction used in this work. (**b**) Density-difference plot highlighting the electronic rearrangement between reactant and product. (**c**) Orbitals used in the active-space construction, ordered according to their contribution to the density difference. The active space is grown from the HONO–LUNO level in an “inside-out” manner.

In Figure 2, we visualize the Active Space Selection process and the respective electronic densities for the system under investigation.

### Quantum-chemical simulations

Once the active-space orbitals are determined at the *Γ*-point, the remaining orbitals are kept outside the correlated active-space treatment as frozen inactive orbitals, and the Born–Oppenheimer Hamiltonian is systematically reduced by projecting it into the active space using standard techniques. We use CCSD calculations of the active-space Hamiltonian, as well as Complete Active Space Configuration Interaction (CASCI) and Heat-bath Configuration Interaction (HCI) calculations, for benchmarking the quantum algorithms^39,40^.

For further refining the HCI results, we apply excitation operators to the final HCI ground state following a method referred to as Extended Heat-bath Configuration Interaction (Ext-HCI). In practice, the system is first diagonalized in the HCI space, and the resulting ground-state amplitudes are then used to define the extension. Configurations with amplitudes below $10^{-2}$ are discarded. For the remaining configurations, all single excitations are generated, while double excitations are included only for configurations with amplitudes above $10^{-1}$. This choice was made empirically to improve accuracy while keeping the memory cost of the extended space manageable. The initial HCI calculations were performed using a varying selection threshold ranging between $10^{-3}$ to $10^{-6}$. For the larger active spaces, these calculations were pushed to the tightest settings that remained feasible within the available computational resources, and the resulting HCI ground state was used as the starting point for the Ext-HCI construction.

**Fig. 3:**
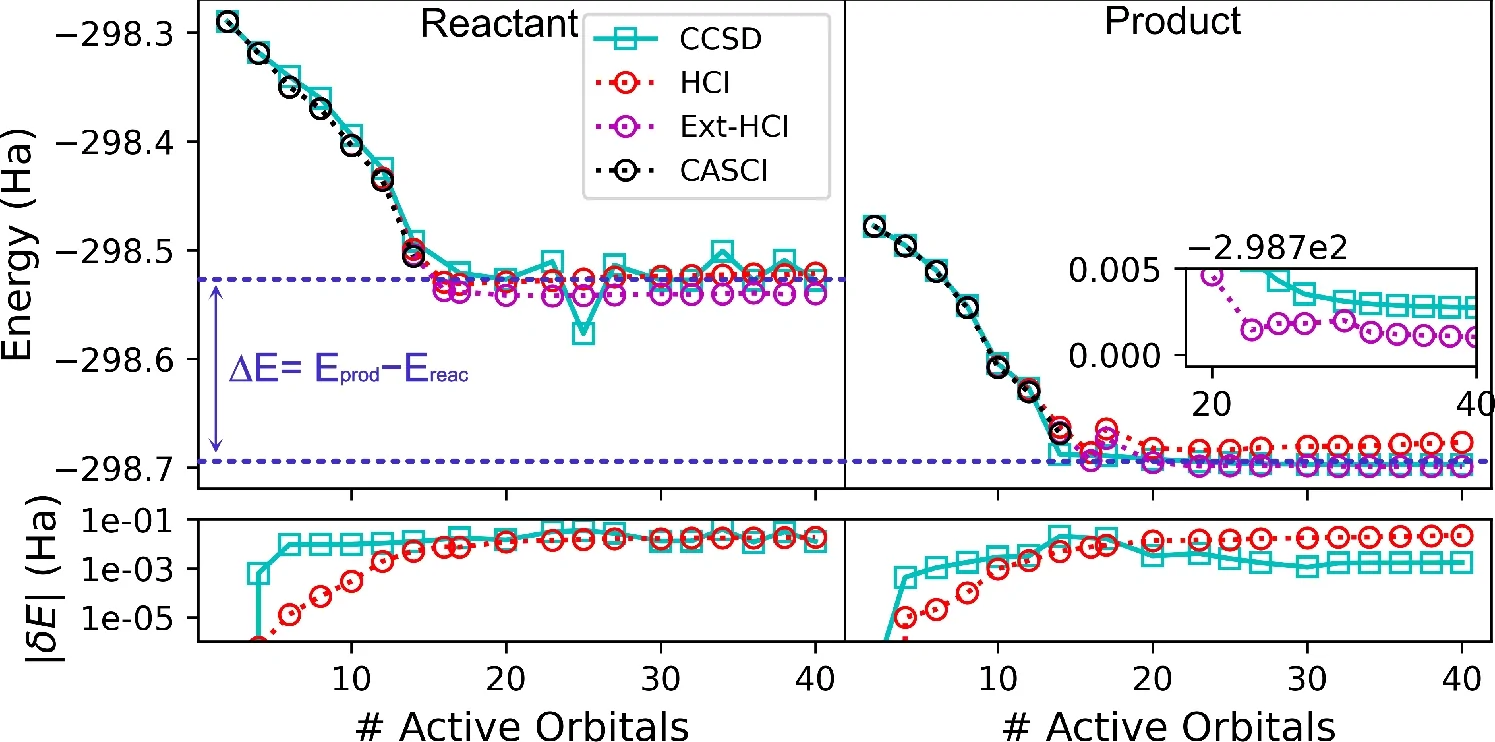
Ground state and surface reaction energies obtained with multiple quantum-chemical simulation methods. Ground state energies as a function of active space size. The curves on the left-hand side represent the reactant, the curves on the right-hand side the product. The blue dashed horizontal lines indicate how reaction energy is calculated, the difference between the product and reactant energy levels defined as $\Delta E = E_{\textrm{prod}} - E_{\textrm{reac}}$. Lower panels show energy deviations relative to Ext-HCI.

In Figure 3, we show the results obtained with the “classical”, quantum-chemical reference methods. Energy values converge at around 20 orbitals and continue to decrease up to 40 orbitals for HCI, Ext-HCI, and CCSD. The results suggest that, for the present system and within the range accessible here, the reaction energy is close to converged by about 40 orbitals, consistent with the near-indistinguishable (within 0.1 mHa) 40- and 50-orbital results shown in Figure 7. However, due to resource limitations, we are not able to scale CASCI calculations beyond 14 orbitals, but we can see reasonable agreement between the other three methods with errors within a few tens of mHa in this range. Therefore, Ext-HCI becomes the standard reference benchmark for higher orbital counts. For this reason, errors are calculated with respect to Ext-HCI, the most accurate, quantum-chemical reference method used in our investigation. As expected, the prediction error increases as we add orbitals to the active space.

### Quantum sampling algorithms

For the quantum experiments, we reformulate the Hamiltonian in second quantization and convert it into a qubit representation by means of fermion-to-qubit transformations using Jordan-Wigner (JW) encoding. SQD then projects the electronic Hamiltonian onto a subspace of sampled electronic configurations, ensuring proper electron count and spin sector. Similar to Quantum Selected Configuration Interaction (QSCI)^41^, SQD systematically expands the subspace for controlled accuracy improvements while maintaining computational efficiency. Our calculations involved *5-10* iterations, with 16 batches per iteration.

**Fig. 4:**
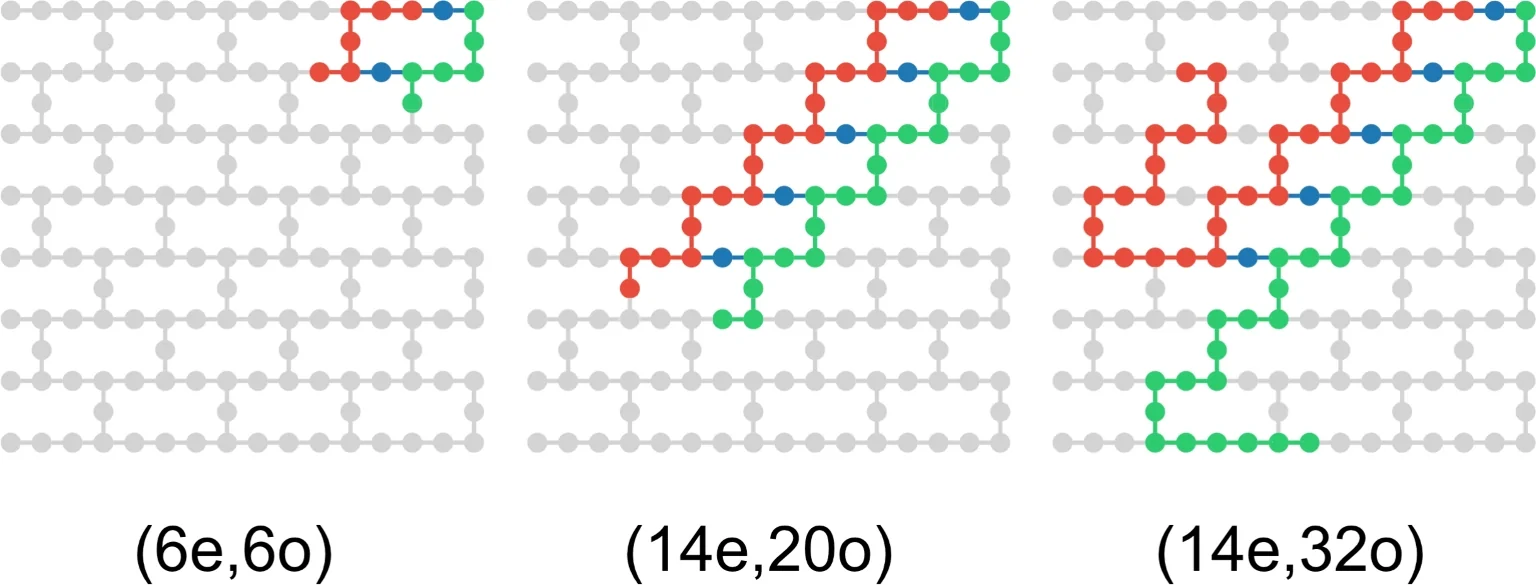
Mapping the LUCJ ansatz onto the quantum computing chip. The LUCJ ansatz represented as a gate based circuit is mapped to the ibm_aachen chip topology. Green qubits represent *α* spin orbitals, red qubits represent *β* spin orbitals, blue qubits denote ancillae present in the heavy-hex implementation of the LUCJ ansatz responsible for *α*-*β* orbital interactions. Once the mapping is made, the quantum circuit undergoes a transpilation step to obtain the final transpiled circuits which are run to sample bitstrings.

To enhance sampling quality, we use a truncated LUCJ ansatz^19^, omitting final orbital rotations and Jastrow interactions from the next layer to balance accuracy and efficiency. The rationale for using the truncated two-layer LUCJ ansatz follows the discussion in the original SQD work^17^, where the effect of the truncated construction on the sampling distribution is explained in terms of the underlying circuit operators. As shown in Figure 4, we initialize the LUCJ ansatz parameters with the one- and two-body amplitudes taken from the CCSD simulations. We build quantum circuits using the ffsim^42^ package and map them to the ibm_aachen chip topology.

**Fig. 5:**
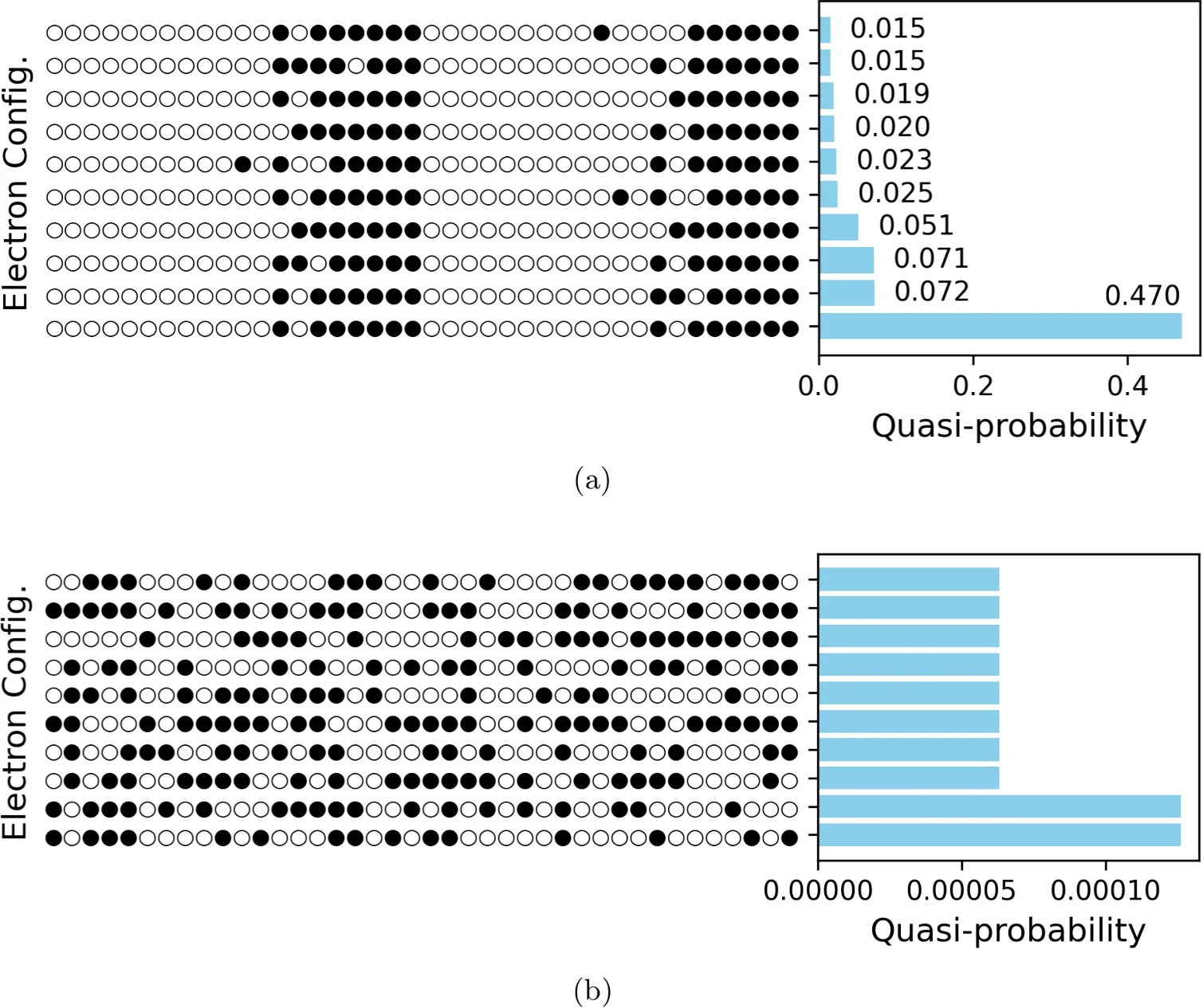
Histograms of the top-10 electron configurations sampled by the quantum processing unit for the (14e,20o) reactant active space. Filled (unfilled) circles represent occupied (unoccupied) orbitals. (a) Histogram for the non-truncated, one-layer LUCJ ansatz. (b) Histogram for the truncated, two-layer LUCJ ansatz. These histograms represent the raw measurement distribution prior to the configuration-recovery step used in SQD. The truncated variant produces a broader distribution that is less dominated by the initial single-determinant reference state, but also contains a larger fraction of bitstrings outside the target particle-number sector.

In Figure 5, we show histograms of the raw output bitstrings produced by the LUCJ circuits for the reactant in the *(14e,20o)* active space, i.e., with 14 electrons distributed over 20 orbitals. In Figure 5a, the non-truncated, one-layer LUCJ ansatz^17^ yields a distribution dominated by the initial single-determinant reference state and its excitations. In Figure 5b, we show the corresponding results for the truncated, two-layer LUCJ circuit. In this case, the raw distribution is less concentrated on the reference configuration and exhibits broader support over electronic configurations.

This choice reflects a tradeoff. The truncated two-layer ansatz produces a broader raw sampling distribution, which can yield a more representative recovered subspace for SQD after configuration recovery. At the same time, it also increases the fraction of bitstrings outside the target particle-number sector, making configuration recovery more challenging. For the purpose of our scaling study, we therefore use the truncated two-layer LUCJ ansatz, since within the regime studied here its broader configurational support proved more useful than the more strongly reference-dominated distribution of the one-layer variant.

**Table 1:** Metrics of representative quantum circuits. *q* is the total number of qubits, which is the sum of *s* qubits representing spin orbitals and *a* ancilla qubits. *d* is the 2-qubit circuit depth, *CX* is the number of CNOT gates and *u* the number of 1-qubit gates.

**System**	*q (s+a)*	*d*	*CX*	*u*
Prod(6e,6o)	14(12+2)	74	132	224
Prod(12e,12o)	30(24+6)	124	474	872
Prod(14e,20o)	48(40+8)	188	1228	2345
Prod(14e,32o)	80(64+16)	466	3026	5551
Reac(6e,6o)	14(12+2)	72	132	222
Reac(12e,12o)	30(24+6)	124	474	870
Reac(14e,20o)	48(40+8)	184	1194	2289
Reac(14e,32o)	80(64+16)	392	2874	5388

In Table 1, we report hardware transpiled circuit sizes and qubit requirements for representative circuits. The circuit size is determined by the depth and number of gates. Our circuit requirements are similar to those reported in Ref.^17^. Note that hardware transpilation will include additional ancilla qubits. We optimized qubit selection based on calibration data using mapomatic^43^, thus excluding qubits with high error rates allowing the mapomatic transpilation workflow to place the circuit in better-connected and less error-prone regions of the device. Empirically, permitting these additional transpilation ancillae led to more favorable measured bitstring distributions for SQD post-processing and improved recovered energies. Each circuit is sampled at most $6 \times 10^6$ times on the ibm_aachen device and results are processed as bitstring distributions for SQD post-processing.

Across the hardware-sampled circuits considered here, the raw bitstring distributions contain on average 93.57% forbidden configurations, i.e., strings with incorrect Hamming weight. This average is taken over all circuits in the study, with smaller active spaces yielding substantially lower forbidden fractions. At the same shot budget, however, the truncated LUCJ ansatz produces on average about 80 times more valid bitstrings with the correct Hamming weight than a uniform random distribution over the same space, for which the average forbidden fraction is 99.922%.

At about 32 orbitals, the circuits become considerably more demanding. With 2935 CNOT gates, we are close to the practical noise limit. In this regime, the average forbidden fraction increases to 96.54% for the reactant and 96.28% for the product, which negatively affects the SQD energy estimates. Practical SQD performance depends on configuration recovery retaining sufficient support in the target particle-number sector. In the present results, the *(14e,32o)* cases lie near the threshold at which the raw sampled data cease to be sufficiently informative for configuration recovery to remain effective, as reflected by the deterioration of the SQD energies at larger active-space sizes. Details are available in Appendix A. To further enhance the accuracy of molecular ground-state energies beyond SQD, we will explore in the following the Ext-SQD method.

Ext-SQD increases accuracy by applying excitation operators to sampled configurations^18^. In direct analogy with the Ext-HCI construction, we discard configurations with amplitudes below $10^{-2}$. For the remaining configurations, we generate single excitations when the amplitude lies between $10^{-2}$ and $10^{-1}$, and both single and double excitations when the amplitude exceeds $10^{-1}$. This procedure enlarges the projected subspace substantially, with the precise growth depending on the amplitude distribution of the preceding SQD or HCI calculation. The resulting subspace dimensions for representative cases are reported in Table 2. Ext-SQD calculations do not rely on an additional configuration-recovery step, since the extension is applied only after the preceding SQD procedure has already produced configurations in the target particle-number sector. The Ext-SQD space is then constructed by applying excitation operators classically to these configurations. We keep the number of batches at 16.

**Fig. 6:**
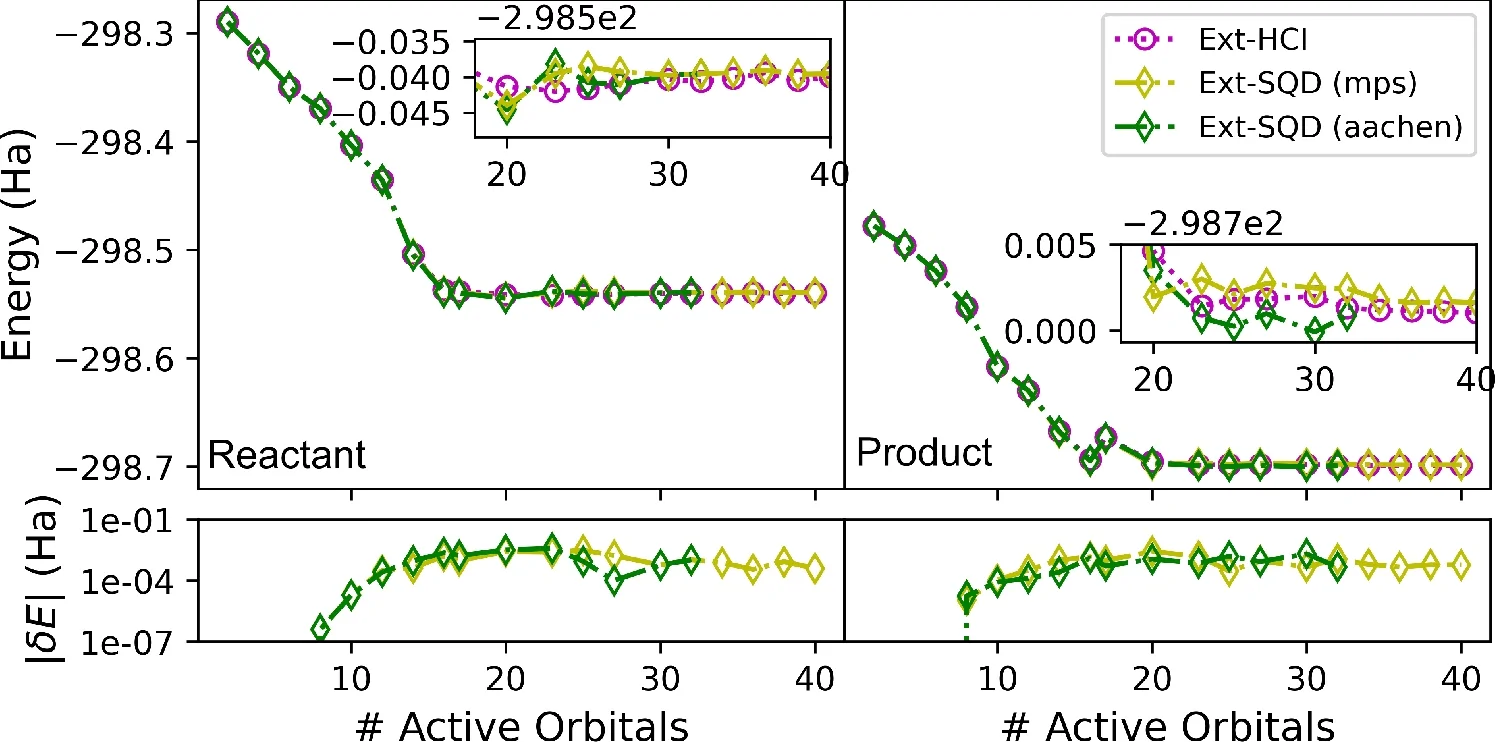
Comparison of ground-state energies obtained with the quantum processing unit. Simulated (yellow diamonds) and experimental (green diamonds) Ext-SQD results are compared against Ext-HCI in the lower panels, the classical reference standard. Ext-SQD experiments can be performed for active-space sizes up to 32 orbitals.

As shown in Figure 6, the Ext-SQD energy predictions achieve higher accuracy than SQD, consistently close to those produced by Ext-HCI. As compared to both HCI and CCSD, Ext-SQD results satisfy the $1.6~\text {mHa}$ chemical accuracy bound. We observe that Ext-SQD reaches lower energy values for both product and reactant above 14 orbitals. Scaling Ext-SQD beyond 32 orbitals is challenging, as the quality of quantum-sampled configurations deteriorates. At and above 34 orbitals, we are unable to obtain configurations with correct Hamming weights within a set of $6 \times 10^6$ samples.

At this scale, as shown in Figure 4, the processor topology limits the mapping of circuits to the lattice diagonals. Adding orbitals makes it increasingly difficult to design circuits that avoid certain qubits and gates with high error rates, reducing sample quality overall.

For scaling SQD and Ext-SQD experiments up to 40 orbitals, we approximate the LUCJ quantum circuits with tensor networks using a truncation value of $10^{-8}$ and Matrix Product State (MPS) simulators^44,45^. While we are indeed able to obtain the results, this approach produces slightly higher energy values, due to the truncation step, as can be seen in Figure 6.

**Fig. 7:**
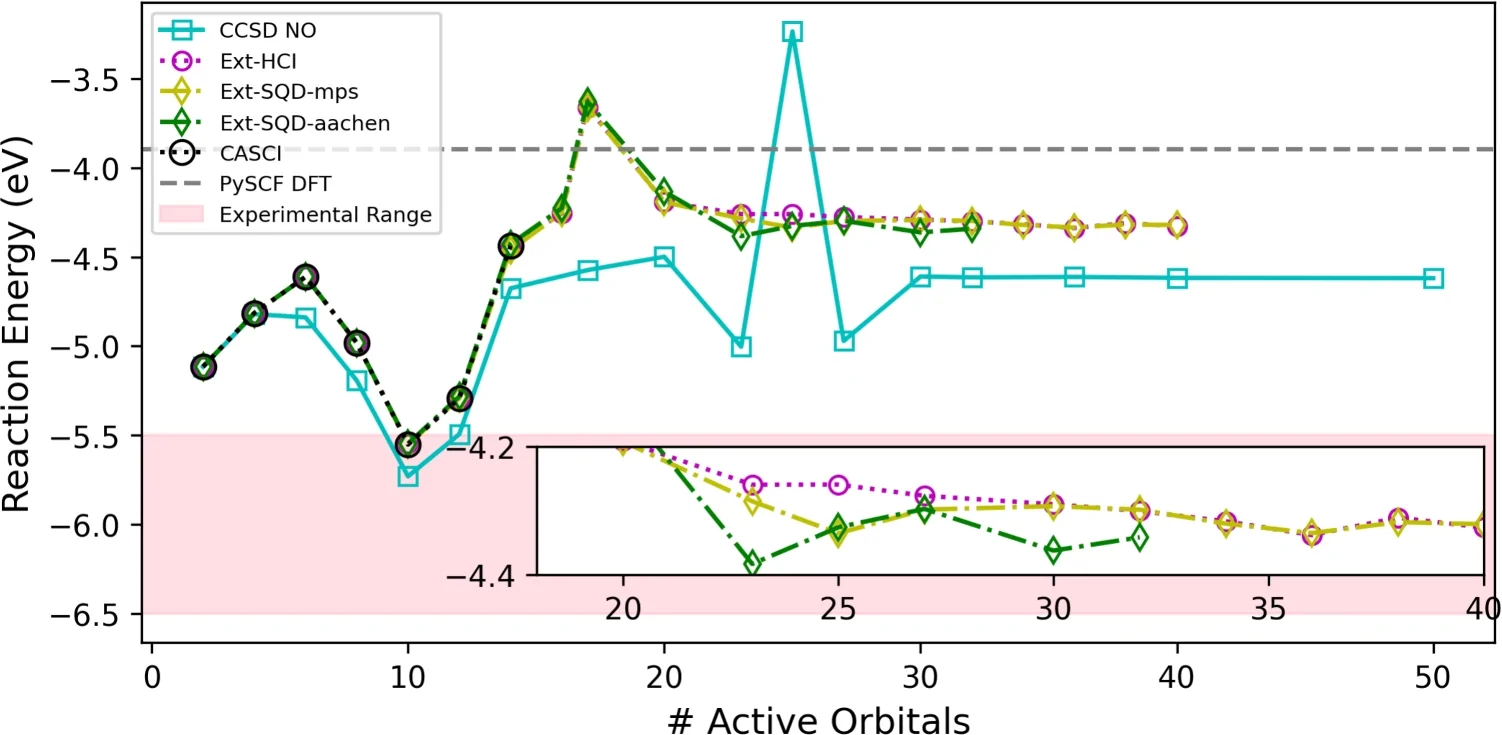
Reaction energies computed at the *Γ***-point.** Reaction energies obtained with quantum and classical methods are compared. DFT results are shown as a reference.

The post-processing calculations are conducted on HPC nodes with 192 x86 cores and 3 TB of RAM by means of the Dice diagonalizer^46^. As shown in Table 2, as the number of orbitals increases, the subspace dimension for Ext-SQD grows faster than for standard SQD. While the subspace dimension for Ext-SQD is consistently smaller than for Ext-HCI, it is still of the same order of magnitude. This is because the number of single excitations grows quadratically with the number of orbitals, while the number of double excitations grows with the fourth power. At higher orbital counts, this limits the number of double excitations that can be included in Ext-SQD^18^.

In Figure 7, we plot the reaction energy for active spaces with up to 50 orbitals and the DFT-values as a reference. Reaction energy values for representative active spaces are provided in Table 3. HCI, Ext-SQD and, CCSD provide lower energy values, closer to those found in literature, which usually rely on empirical corrections^36–38,47^. While we are unable to consistently scale beyond 40 orbitals, the energy values produced with Ext-HCI and Ext-SQD exhibit convergence at smaller active space sizes than in the case of CCSD. While CCSD provides a *Δ E* value closer to experimental references, its individual reactant and product ground-state energies are not as favorable as those obtained with the variational CI-based approaches. This indicates that the improved reaction energy could have come from partial cancellation of errors between the two total energies, rather than from uniformly better absolute-energy predictions. We note that CCSD, unlike CI based methods such as CASCI, HCI, Ext-HCI, SQD and Ext-SQD^17,41^, is not a variational method. This means it can predict values below the actual ground state energy^48,49^, which complicates the comparison.

Using atomic coordinates derived from DFT relaxations, all subsequent prediction methods are expected to be affected by the mean-field nature of DFT in defining the reactant and product states. Nevertheless, the prediction results improve upon the initial reaction energy values overall, successively approaching the experimental range.

**Table 2:** Ext-SQD and Ext-HCI calculations for product and reactant in representative active spaces. $|\tilde{\chi }|$ is the number of samples taken from **ibm_aachen**, *K* is the number of configuration recovery iterations performed in SQD.*D*, $D_E$, and $D_{EH}$ denote the effective projected-space sizes used in SQD, Ext-SQD, and Ext-HCI, respectively. These quantities are computed as the product of the retained *α*- and *β*-configuration space dimensions.

System	$|\tilde{\chi }|$	*K*	*D*	$D_{E}$	$D_{EH}$
Prod(2e,2o)	$1.5\cdot 10^{6}$	5	4	4	4
Prod(12e,12o)	$1.5\cdot 10^{6}$	7	$3.4\cdot 10^{5}$	$2.0\cdot 10^{5}$	$2.1\cdot 10^{5}$
Prod(14e,20o)	$2.0\cdot 10^{6}$	10	$1.4\cdot 10^{6}$	$8.0\cdot 10^{6}$	$8.2\cdot 10^{6}$
Prod(14e,30o)	$2.0\cdot 10^{6}$	10	$1.4\cdot 10^{6}$	$4.4\cdot 10^{7}$	$8.0\cdot 10^{7}$
Reac(2e,2o)	$1.5\cdot 10^{6}$	5	4	4	4
Reac(12e,12o)	$1.5\cdot 10^{6}$	7	$3.4\cdot 10^{5}$	$1.8\cdot 10^{5}$	$4.7\cdot 10^{5}$
Reac(14e,20o)	$2.0\cdot 10^{6}$	10	$1.3\cdot 10^{6}$	$9.0\cdot 10^{6}$	$1.1\cdot 10^{7}$
Reac(14e,30o)	$2.0\cdot 10^{6}$	10	$1.3\cdot 10^{6}$	$2.8\cdot 10^{7}$	$5.6\cdot 10^{7}$

**Table 3:** Comparison of reaction energies obtained in representative active spaces. The *Δ E*-values are provided in eV.

**Act. Space**	**CASCI**	**CCSD**	**Ext-HCI**	**Ext-SQD (MPS)**	**Ext-SQD (aachen)**
(2e,2o)	*-5.116*	*-5.116*	*-5.116*	*-5.116*	*-5.116*
(12e,12o)	*-5.294*	*-5.494*	*-5.294*	*-5.294*	*-5.283*
(14e,20o)	N/A	*-4.500*	*-4.191*	*-4.193*	*-4.134*
(14e,30o)	N/A	*-4.610*	*-4.290*	*-4.293*	*-4.363*
(14e,32o)	N/A	*-4.615*	*-4.301*	*-4.299*	*-4.342*

## Discussion

The results presented in this study demonstrate the potential of hybrid quantum-classical methods for improving the accuracy of electronic structure calculations. By leveraging a systematic active-space selection approach and employing SQD and Ext-SQD, respectively, we obtain ground-state energies that, in selected active-space regimes, are comparable to or lower than Ext-HCI. Since Figure 3 establishes Ext-HCI as the strongest classical method among the ones considered, we use it as the primary classical reference in Figure 6. The agreement of *Δ E* results obtained with Ext-SQD and CASCI for active spaces up to 12 orbitals, along with the scaling of Ext-SQD up to 32 orbitals, demonstrates the utility of the approach for studying electronic configurations in chemistry and materials science.

We note that the differences between the energy values obtained with Ext-SQD and Ext-HCI are small enough to be considered chemically equivalent, i.e., *<1.6* mHa, while maintaining similar subspace dimensions, see Table 2. The same applies to the calculated reaction energies.

The methodology outlined in Fig. 1 integrates classical and quantum techniques to achieve computationally efficient simulations. DDA identifies the chemically relevant active region, while the subsequent use of coupled-cluster natural orbitals refines the orbital representation within that space. In this way, the active space retains the electronic degrees of freedom most relevant to the reaction, while the natural-orbital step improves the compactness, convergence, and accuracy of the correlated calculations, consistent with the DD+NO strategy introduced in prior work^7^.

The integration of quantum hardware in the computational architecture allowed us to validate the approach with a state-of-the-art quantum processor. As shown in Table 1, circuit depths and gate counts increase significantly with the number of orbitals in the active space, limiting the upscaling of quantum simulations with current quantum processing units. The results obtained from executing LUCJ circuits confirm that our approach is feasible on current quantum devices, but noise mitigation strategies will be necessary to maintain accuracy at larger system sizes. Importantly, the simulation results indicate the feasibility of extended active spaces, beyond 32 orbitals, as soon as quantum processing units with lower error rates become available.

As a key finding of our investigation, Ext-SQD provides significant improvements over standard SQD by incorporating excitation operators to refine the sampled configuration space. The enhancement allows for better representation of ground-state wavefunctions while maintaining computational feasibility. While Figure 6 shows the energy trends with increasing active-space size, Table 2 shows that the effective projected-space size in Ext-SQD grows substantially with the number of orbitals, increasing the cost of the classical post-processing and shifting the practical bottleneck from quantum sampling to classical resources. This behavior can also be observed in the comparison between HCI and Ext-HCI at a smaller scale, where the corresponding energy values deviate increasingly from one another for active spaces above 14 orbitals. Research and development is needed for optimizing classical post-processing techniques, such as parallelizing the diagonalization of extended subspaces. Such improvements could unlock scaling up to 40 orbitals, where the computational bottleneck shifts back to the quantum stack.

An important result of our scaling study is the rigorous comparison with classical methods as function of active space size. Based on the analysis in Figure 7, we monitor how the energy differences computed with Ext-SQD converge earlier than those obtained with CCSD. For the present system, the 20-orbital calculation consistently provides a particularly favorable balance between subspace quality and computational cost within the current computational infrastructure, yielding Ext-SQD energies below those obtained with CCSD and HCI across repeated runs of the workflow. As shown in Figure 7, this is also the regime in which the Ext-SQD reaction energies begin to, in some cases, slightly improve upon, those obtained with Ext-HCI. This indicates that quantum methods, when properly integrated with classical techniques, can deliver improvements of both computational efficiency and accuracy with regard to classical reference methods.

So far, we have applied variational references (HCI/Ext-HCI, SQD/Ext-SQD) and CCSD in the active space. A natural question is the role of dynamic correlations outside of the active space. Established, multi-reference second-order perturbation theories, e.g., CASPT2 and NEVPT2, are designed to recover such correlation in CAS-quality^50,51^. In practice, CASPT2 often employs level-shift strategies, real or imaginary, and the IPEA shift to mitigate intruder states^52,53^, while NEVPT2 is intruder-state free and rigorously size-consistent in its contracted formulations^51^.

From an algorithmic standpoint, our Ext-SQD wavefunctions provide multi-reference states within the active space for defining a subsequent PT2 correction over external orbitals. This mirrors the SCI+PT strategy used with selected-CI references^54,55^, and offers a route to incorporate external-space, dynamic correlation without increasing quantum circuit depth. The net effect likely depends on differential, external correlations between reactants and products. Although we do not report CASPT2/NEVPT2, extending Ext-SQD with PT2 and NEVPT2 is, therefore, a plausible direction for scaling accuracy.

Despite the advancements, several challenges must be addressed before quantum-assisted simulations become a practical tool for chemistry and materials applications. Noise resilience, circuit depth limitations, and efficient state preparation remain key obstacles. The LUCJ ansatz, while effective in reducing circuit depth, may require further modifications to improve expressivity and error resilience. Moreover, exploring alternative quantum ansatzes, such as adaptive variational circuits, could improve energy estimations while maintaining hardware efficiency.

Looking ahead, the insights gained from this work provide a strong foundation for future research in quantum-assisted simulations of surface reactions. The extension of our approach to more complex reaction networks, multi-step catalytic processes, and beyond lithium-oxygen systems will be an important direction. As quantum hardware continues to advance, the scalability of hybrid methods like SQD and Ext-SQD will be further tested,

In practice, they are determined by both the sampling cost on the HW and the classical post-processing stage. Sufficient measurements or shots are needed to construct a distribution of bitstrings from which sub-spaces are further sampled and refined using configuration recovery. Additionally, the cost associated with classical diagonalization of the projected Hamiltonian can increase steeply as the subspace dimensions grow. For Ext-SQD in particular, this classical cost depends strongly on the excitation-expansion strategy. If the extension is applied too aggressively, the projected space can grow rapidly; however, this growth remains tunable through the amplitude thresholds and the choice of excitation classes, allowing the balance between accuracy and computational cost to be controlled. More in depth discussions on scalability can be found in the original works^17,18^. It is also important to note that recently significant progress has been made to reduce the cost of classical diagonalization by introducing highly parallelized GPU accelerated codes^56^.

In conclusion, this study demonstrates that hybrid quantum-classical methodologies, particularly those leveraging advanced active space selection and sample-based diagonalization techniques, open a promising path for studying complex chemical problems. Our results underscore the potential of quantum computing to complement and extend classical approaches, bringing us closer to achieving accurate and efficient electronic structure calculations in materials discovery.

## Methods

### DFT simulations

Following the procedure outlined in reference^7^, after performing a geometry optimization step, we conducted DFT calculations with a basis of translationally adapted, linear combinations of Gaussian atomic orbitals (AOs) which are expressed as:

1 $$\begin{aligned} \phi _{k,p}(r) = \sum _{T} e^{i k \cdot T} \chi _p (r - T), \end{aligned}$$

where $T = \sum _{i=1}^{3} T_i a_i$ represents a lattice translation vector, $k = \sum _{i=1}^{3} k_i b_i$ denotes a momentum vector within the first Brillouin zone and $\chi _p$ is a Gaussian-type atomic orbital. The summation over *T* ensures that the constructed orbitals conform with the translational periodicity of the system^57,58^.

All electronic structure calculations were performed using the PySCF package^34^, employing the GTH-DZV basis set^32^and the Goedecker-Teter-Hutter (GTH) pseudopotentials^32^, as well as the Perdew-Burke-Ernzerhof (PBE) exchange-correlation functional^33^.

Within this basis, the Born-Oppenheimer Hamiltonian takes the following representation^59^:

2 $$\begin{aligned} \begin{aligned} \hat{H} \;=\;&\, E_0 + \sum _{\begin{array}{c} \textbf{k}\\ p\,r\,\sigma \end{array}} h_{pr}(\textbf{k})\, \hat{a}^\dagger _{\textbf{k} p \sigma }\, \hat{a}_{\textbf{k} r \sigma } \\&\;+\; \frac{1}{2}\, \sum _{\begin{array}{c} \textbf{k}_p,\textbf{k}_r,\textbf{k}_q,\textbf{k}_s\\ p\,r\,q\,s\,;\,\sigma \,\tau \end{array}}^{\!*} \big (\,\textbf{k}_p p,\;\textbf{k}_r r \,\big |\, \textbf{k}_q q,\;\textbf{k}_s s\,\big )\, \hat{a}^\dagger _{\textbf{k}_p p \sigma }\, \hat{a}^\dagger _{\textbf{k}_q q \tau }\, \hat{a}_{\textbf{k}_s s \tau }\, \hat{a}_{\textbf{k}_r r \sigma }\,, \end{aligned} \end{aligned}$$

where momentum conservation is enforced such that $k_p + k_q - k_r - k_s = G$, with *G* being a reciprocal lattice vector. To efficiently approximate electron-electron interactions, we employed density fitting with a Weigend auxiliary basis.

### Automated active space selection

We localized the Kohn-Sham orbitals using the Pipek-Mezey method^60^, which relies on a Mulliken population analysis with meta-Löwdin orbitals^61,62^. Localization was performed separately for occupied and virtual orbitals at each individual **k**-point to ensure that the electronic structure characteristics were preserved across the Brillouin zone. We computed the electronic density difference at the DFT level, as defined by

3 $$\begin{aligned} \rho _{\text {DD}}(\textbf{x}) = \rho _{\text {Li}+\text {O}_2}(\textbf{x}) -\rho _{\text {O}_2}(\textbf{x}) - \rho _{\text {Li}}(\textbf{x}), \end{aligned}$$

which quantifies the charge redistribution during the reaction, as shown in Figure 2b.

To automate the selection of active spaces, we ranked orbitals based on their contributions to the density difference. To quantify these contributions, we introduced a threshold parameter *η* for suppressing minor orbital components and for retaining only physically meaningful orbitals. By systematically varying this threshold, we identified a range where orbital rankings remain consistent, facilitating a robust active space selection. Through a systematic scan, we identified a stability range of $\eta \approx 10^{-3}$. Within this range, the ranking of occupied localized orbitals remained stable.

To further enhance active space selection and ensure convergence with respect to active space size, we combined the density difference analysis with natural orbitals obtained from CCSD calculations. Specifically, an initial CCSD calculation was performed within an active space constructed from the highest-ranked occupied orbitals identified via the density difference analysis and all virtual orbitals. The resulting natural orbitals exhibit occupation numbers predominantly close to either zero or two, clearly distinguishing high-occupancy orbitals from low-occupancy ones. In cases involving stronger electron correlation, orbitals with fractional occupancy had to be explicitly included. The final active space was systematically constructed around the Highest Occupied Natural Orbital (HONO) and the Lowest Unoccupied Natural Orbital (LUNO). For further detail, we refer to reference^7^.

### Sample-based quantum diagonalization

The Sample-based Quantum Diagonalization (SQD)^17,41,63^ approach involved projecting the electronic Hamiltonian onto a subspace defined by a collection of electronic configurations sampled from a probability distribution. These set of configurations, denoted as $X = \{x\}$, were drawn from the wavefunction probability $P_{\Psi }(x) = |\langle x | \Psi \rangle |^2$, whereas a noisy version is represented by $P_{\tilde{\Psi }}(x) = \langle x | \tilde{\rho } | x \rangle$. The subset of configurations satisfying the target particle-number constraints was labeled $X_N$, and a self-consistent configuration-recovery procedure was then used to construct an expanded recovered set $X_R$ consistent with the target particle-number and spin sector. The electronic Hamiltonian $\hat{H}$ was then projected onto the selected subspace, and its low-energy eigenvalues and eigenvectors wer determined via direct diagonalization using the iterative Davidson algorithm^64^ across multiple classical computing nodes^17^.

To obtain the set of configurations, we sampled a suitable circuit ansatz on a quantum device. The Local Unitary Cluster Jastrow (LUCJ)^19^ ansatz is a variational quantum circuit designed to generate approximate ground states of electronic systems while maintaining a moderate circuit depth. It is derived from unitary coupled-cluster (UCC) theory and incorporates local approximations to reduce the number of required two-qubit gates. The LUCJ wavefunction is constructed as

4 $$\begin{aligned} |\Psi \rangle = \prod _{\mu =1}^{L} e^{\hat{K}_\mu } e^{i \hat{J}_\mu } e^{-\hat{K}_\mu } |x_{\text {ref}}\rangle , \end{aligned}$$

where $\hat{K}_\mu = \sum _{pr,\sigma } K^\mu _{pr} \hat{a}^\dagger _{p\sigma } \hat{a}_{r\sigma }$ are one-body excitation operators, $\hat{J}_\mu = \sum _{pr,\sigma \tau } J^\mu _{p\sigma , r\tau } \hat{n}_{p\sigma } \hat{n}_{r\tau }$ are density-density interactions and $|x_{\text {ref}}\rangle$ represents the reference Hartree-Fock state encoded in the Jordan-Wigner mapping.

The circuit depth can be further reduced by employing a truncated version of the LUCJ ansatz. Specifically, we used the form

5 $$\begin{aligned} |\Psi \rangle = e^{\hat{K}_2} e^{-\hat{K}_1} e^{i \hat{J}_1} e^{\hat{K}_1} |x_{\text {ref}}\rangle . \end{aligned}$$

which represents to a two-layer LUCJ circuit, in which the final orbital rotation $e^{\hat{K}_2}$ and Jastrow interaction $e^{i \hat{J}_2}$ are omitted. This reduces the circuit depth to that of a single-layer LUCJ^17^.

In practice, samples obtained from noisy quantum devices do not always satisfy the particle-number and spin constraints of the target electronic problem. For this reason, SQD does not rely exclusively on the subset $X_N$ of sampled configurations with the correct particle number. Instead, it applies a self-consistent configuration-recovery procedure that uses information from the noisy sampled distribution to probabilistically reconstruct configurations consistent with the target $(N_\alpha ,N_\beta )$ sector, thereby generating an expanded recovered set $X_R$. This recovery step is essential for mitigating sampling errors and enlarging the effective subspace used for Hamiltonian projection. Its effectiveness, however, depends on the raw sampled distribution retaining sufficient support near the physically relevant sector; when the sampled distribution is overwhelmingly dominated by configurations with incorrect particle number, the recovery procedure becomes significantly less effective.

Following the standard SQD workflow, we perform configuration recovery iterations to correct bitstrings obtained from the hardware with the wrong particle number. In each iteration, the recovered bitstrings are sub-sampled into multiple batches, and each batch is used to define an independent projected Hamiltonian problem that is diagonalized independently. The batch results are then used to update the average orbital occupancies for the next configuration-recovery iteration, while the lowest-energy batch solution provides the best energy estimate for that iteration. Full algorithmic details are given by Robledo-Moreno et al.^17^.

We employed an extension of the SQD method, termed Extended SQD (Ext-SQD), to increase accuracy of molecular ground-state energies. Ext-SQD incorporates elements of Quantum Subspace Expansion while maintaining the computational efficiency of SQD. In contrast to standard SQD, which forms excited states by providing multiple eigenpairs of a projected Hamiltonian, Ext-SQD expands the accessible configuration space by explicitly applying excitation operators to the computational basis states in the original subspace.

### Compressed circuit simulation via MPS truncation

To access larger active spaces, we approximate the LUCJ circuits using matrix product state (MPS) simulations, following the tensor-network compression strategy described in Ref^44^.. In this representation, the many-qubit wavefunction is compressed by expressing it as a chain of low-rank tensors, which can be much more efficient for circuits with limited entanglement. During the simulation, the application of quantum gates generally increases the bond dimension of the MPS. To keep the calculation tractable, the state is compressed after gate applications by discarding Schmidt coefficients below a chosen truncation threshold.

In this work, we use a truncation threshold of $10^{-8}$. In the Qiskit Aer MPS backend, this threshold controls the truncation of the Schmidt spectrum by discarding the smallest Schmidt coefficients whose total squared weight is below the specified value. Smaller thresholds retain more Schmidt coefficients and therefore provide a more faithful representation of the circuit output state, at the expense of higher computational cost, whereas larger thresholds yield stronger compression but introduce a larger approximation error. The resulting MPS therefore provides a controlled approximation to the full circuit output state^65^.

For our simulations, LUCJ circuits were generated from coupled-cluster amplitudes and simulated using the MPS backend of Qiskit Aer^65^ in a noiseless scenario. The resulting bitstring samples were then used in the same SQD and Ext-SQD post-processing workflow as for the hardware-generated data.

Unlike Ref^44^., we did not employ a variational optimization routine or fidelity cost function to construct a new parameterized circuit. Instead, we relied on the intrinsic compressibility of the circuit output and the effectiveness of tensor network simulation for shallow-depth, structured circuits. The resulting MPS representations bypassed the need for quantum hardware execution and enabled scalable generation of bitstring samples from variational states for use in SQD post-processing.

## Figures and Tables

**Fig. 8:**
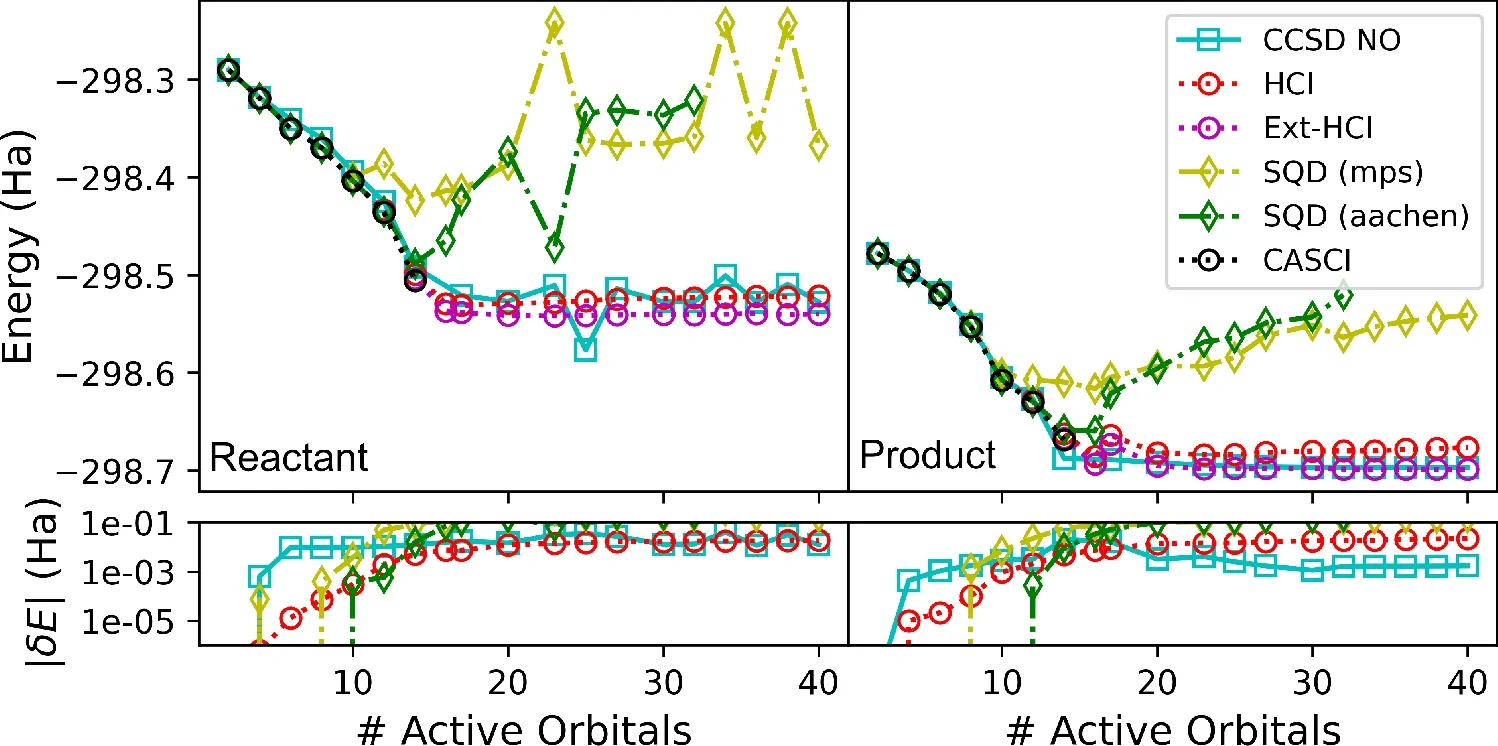
Comparison of ground state and reaction energy results for SQD methods. SQD calculations of the ground state energies as a function of active space size. The left curves are for the reactant and the right ones are for the product. The plotted energies are compared against Ext-HCI, with the lower panel showing absolute differences.

## Data Availability

The datasets used and/or analyzed during the current study are available from the corresponding author on reasonable request.

## Appendix A SQD results

We provide the SQD results for both reactant and product in Figure 8.

## Code availability

Open-source code developed for this work is available in the SQD_battery_reactions repository^66^. The repository contains the workflow used in the paper, including scripts for DFT preprocessing and Pipek–Mezey localization, density-difference and orbital-overlap analysis for active-space selection, CCSD calculations for pairwise and natural-orbital active spaces, Hartree–Fock integral transformations, truncated-LUCJ circuit generation and sampling, SQD/Ext-SQD workflows with DICE, and HCI/Ext-HCI calculations on classical hardware. It also includes the XYZ input structures, Ray-cluster utilities for distributed runs, a PT2 solver compatible with the DICE-based workflow^66^. Open source-code repositories used in this work: PySCF^34^, ffsim^42^, Qiskit SDK^65^, qiskit-addon-sqd^67^, qiskit-addon-aqc-tensor^45^, mapomatic^43^ and Ray^68^. In addition, we used the Vienna Ab initio Simulation Package (VASP)^29–31^.
